# How to Resolve the Maximum Valuable Information in Complex NIR Signal: A Practicable Method Based on Wavelet Transform

**DOI:** 10.3389/fchem.2022.812567

**Published:** 2022-04-07

**Authors:** Jing Chen, Xiaoquan Lu

**Affiliations:** Key Lab of Bioelectrochemistry and Environmental Analysis of Gansu, College of Chemistry and Chemical Engineering, Northwest Normal University, Lanzhou, China

**Keywords:** wavelet transform (CWT), near infrared spectrum, uninformative variable elimination, residual error sum of square, root mean square error

## Abstract

A key problem in the field of near infrared (NIR) spectrum study is to obtain the valuable information from the complex NIR signal. A maximum information extraction method based on Wavelet Transform (WT) is proposed in this paper for helping the relative researchers to resolve the signal. The results show that the method can serve as an effective tool for obtaining the maximum valuable information in NIR study.

## Graphical Abstract

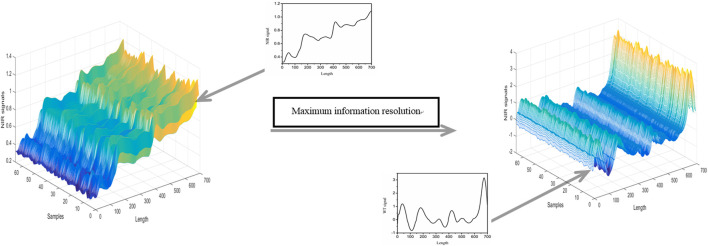

## Introduction

With the advantages of nondestructive measurement, rapidity and simplicity, near-infrared (NIR) spectroscopy has been widely applied to measure samples in the industries of food (Stenlund et al., 2009) and pharmaceutical (Abrahamsson et al., 2005) and the agricultural products (Pedro and Ferreira, 2005; Ozaki et al., 2006). However, the spectral signals of samples which are interfered by background and noise are always seriously overlapping and contain some variations irrelevant to concentration (Rutan et al., 1998). The key problem is how to extract valuable information from these complex spectral bands in the NIR region.

Multivariate calibration models which have been successfully applied to analyze NIR spectral data have greatly developed NIR applications (Inácioa et al., 2013; de Oliveira et al., 2014; Goodarzi et al., 2015; Pan et al., 2015; Yun et al., 2015; Eskildsen et al., 2016). A reliable calibration model is created by sufficient spectral data to assure the predicting accuracy of test set. The weaker the analytical signal of calibration and prediction set is, the worse the model’s predicting accuracy is. Some efforts have been explored to squeeze the complex NIR signal by eliminating “uninformative” signal points. Among them, uninformative variable elimination (UVE) (Centner et al., 1996) has been successfully applied as an classical method. A “stability” is defined in the method to estimate the significance of each signal point, and a cut-off threshold is generated by regression coefficients based on a random variable matrix with small amplitude. Many “uninformative” signal points are eliminated according to this cut-off threshold value. There is the strong possibility to miss some significant signal points because the signal is overlapped seriously.

As an effective mathematical microscope, Wavelet Transform (WT) is very helpful for enlarging the signal details. Here, it is used to extract the maximum information by resolving the original spectrum signal. Then, the signal is reconstructed by the resolved signal before constructing model. The method is a valuable tool for the relative researchers.

## Methods

### Theory and Algorithm

The Continuous Wavelet Transform (CWT) of the signal (or data) *f*(*x*) is defined as:

$$W\left(a,b\right)=\frac{1}{\sqrt{a}}\int_{-\infty }^{\infty }f\left(x\right)\psi^{*}\left(\frac{x-b}{a}\right)dx$$ (1)

**FIGURE 1:**
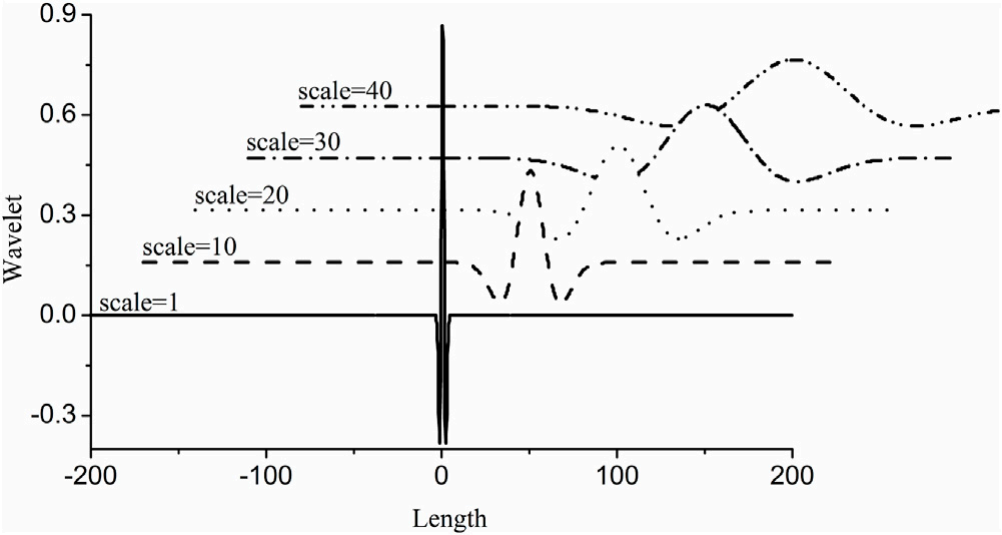
The mexh wavelet with the increasement of *a* from 1 to 40.

Where *W*(*a*,*b*) is the CWT of *f*(*x*), *a* (*a*>0; *a*∈R) is the scaling factor, *b* (*b*∈R) is the window factor, and $\psi^{*}$ is the wavelet which is the dilation and translation of the mother wavelet (Chau et al., 2004; Kalteh, 2013; Subaie and Mourou, 2013; Yuan et al., 2014; de Yong et al., 2015; Martyna et al., 2015; Yu et al., 2015). With the progressive increasement or decreasement of the scale, the wavelet changes regularly. As shown in Figure 1, with the increasement of *a* from 1 to 40, the Mexh wavelet becomes shorter and wider.

It has been widely confirmed that the WT can resolve valuable information in the signal, such as the resolution of overlapping peaks and the cancellation of background and noise (Dinc et al., 2006; Jena et al., 2014; Fu et al., 2015; Lopes-dos-Santos et al., 2015; Dinç and Yazan, 2018). WT is actually the convolution of the initial signal *f(x)* and a special wavelet at a scale value. Since the scale *a* can be a series of consecutive integers (PATHAK and SINGH, 2016), the WT results of the initial signal are spread into a three dimensional space to show the signal details more clearly.

When the wavelet maximum overlaps with the signal at a signal point, the convolution result maximum presents the point information. Our WT program obtained the same results with some commercial softwares. If the scale *a* is set as a fixed value, the wavelet cannot usually maximum overlap with the whole signal at each point. However, in a scale range, the wavelet can maximum overlap with each signal point by the change of wavelet. Therefore, the maximum and minimum WT value of the signal in a scale range are used here to reconstruct signal to present the maximum information at each signal point. The complex NIR signal will be used here. The troughs in the signal may contain some important information. So, the minimum WT values at some signal points are also considered.

### Calculation Methods

For exploring the detailed information of a signal obtained by WT, the methods like WT, UVE and other analytical calculation method are develop, and the simulated signal was generated with Matlab which also has a WT command set integrated in the software. The figures are drew by Origin.

## Results and Discussion

**FIGURE 2:**
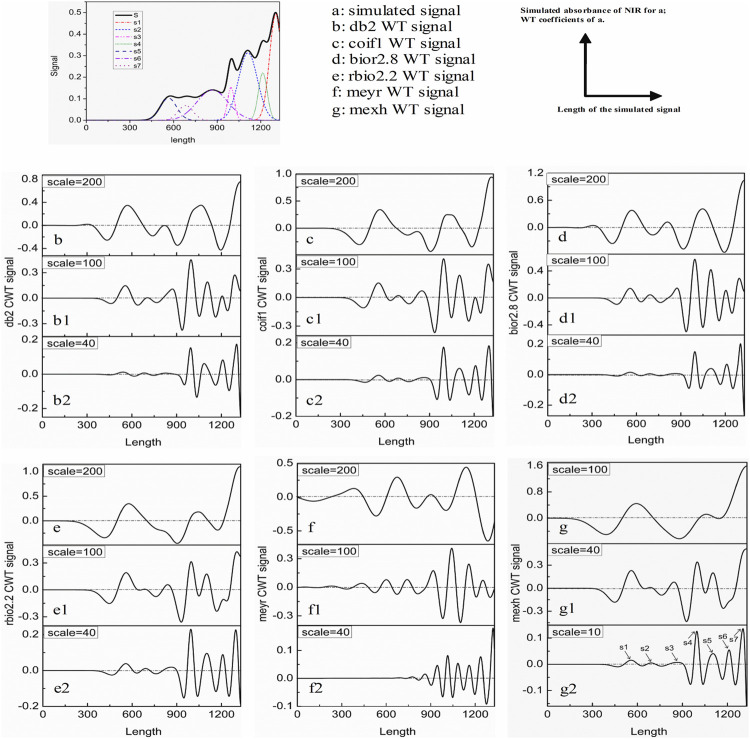
a: the simulated signal S; b–g: the WT results of S by different wavelets with different scales.

The signal S in Figure 2 is simulated by referring the actual NIR spectrum data (Shao et al., 2010) (http://www.idrc-chambersburg.org/shootout2010.html) to show the resolution ability of the method. The simulated signal S is formed by the signals a-g. If there were no effective methods to resolve the simulated signal, it will be easy to lose some valuable information (a-g), and no benefit to qualitative and especially quantitative analysis.

**FIGURE 3:**
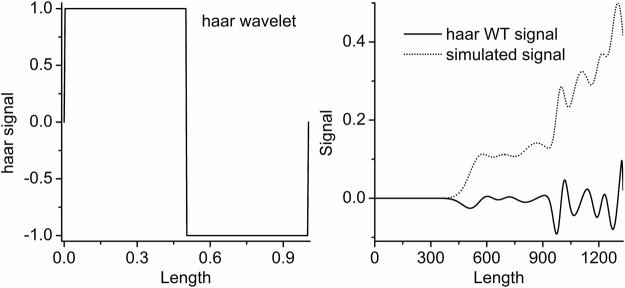
Haar wavelet and the Haar WT result of the simulated signal.

**FIGURE 4:**
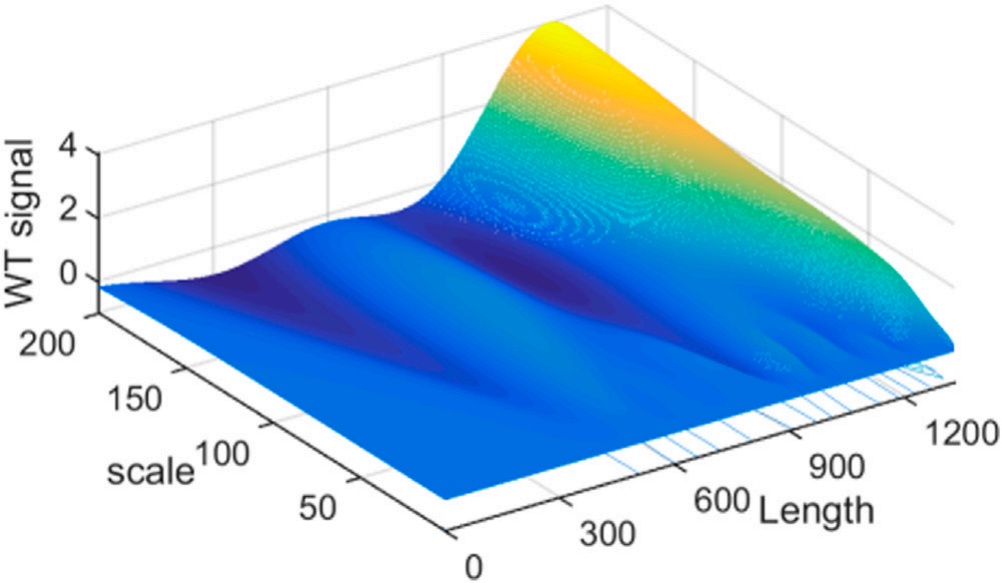
The three dimensional mexh WT figure of the simulated signal.

Haar wavelet can be used to resolve the overlapped signal (Chen et al., 2015). However, it is easy to result in an error qualitative analysis result. That is because Haar WT is same as the first derivation of the signal. The transformed results of the peaks and troughs are zero (Figure 3). In order to assure the ability of intuitive and accurate qualitative and quantitative analysis of the method, the wavelets can obtain the WT results like the second derivative results of the signal are utilized. Figure 2. b–g show the resolution ability of some wavelets at different scales. All WT results are obtained by boundary extension. As is known that the WT results present the background and noise of the signal when the scale *a* is set as a small value, because a higher and narrower wavelet is easy to overlap the subtle background and noise, such as Figure 1. *a* = 1. But some valuable information is easy to be neglected if the scale *a* is set as a too large value. It can be seen from Figure 2. b–g or Figure 4. Therefore, we just select the maximum and minimum WT values in some scale range. Our aim is to afford a useful method to the relative researchers. So, we compared the resolution ability of the wavelets in Figure 2. The relative researchers can select suitable wavelet according to them.

**FIGURE 5:**
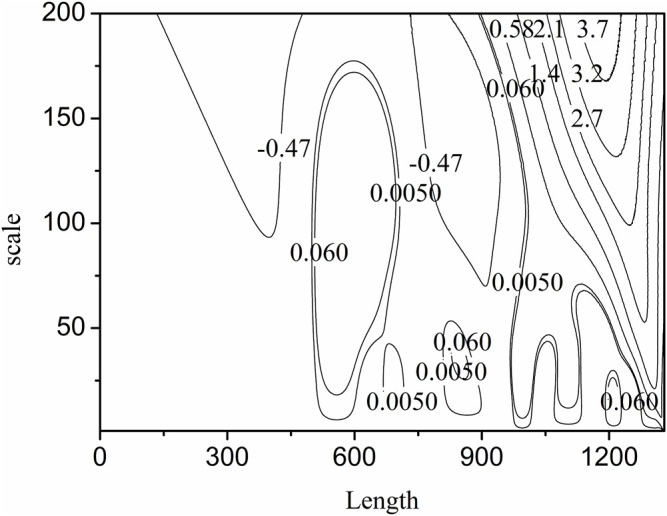
The contour figure of the mexh WT results of the simulated signal.

Figure 2. g is the Mexh WT of the simulated signal. It is same as the second derivation of the signal. It is clear that some information are cancelled when the scale is set as a larger value 100, such as the sub signal s2, s3, s4 and s6. If the scale is set as a suitable value such as 10, all the valuable information can be resolved. By examining the three dimensional Figure with the scale less than 40, if the scale is set as a certain value, some sub signal points maybe occur the maximum WT values, but it is not for others. This is also clear in the contour Figure 5. We just show some sections in this figure. From above analysis, the maximum WT values of the signal in the scale range 40 or some near value can present the maximum information of sub signals.

**FIGURE 6:**
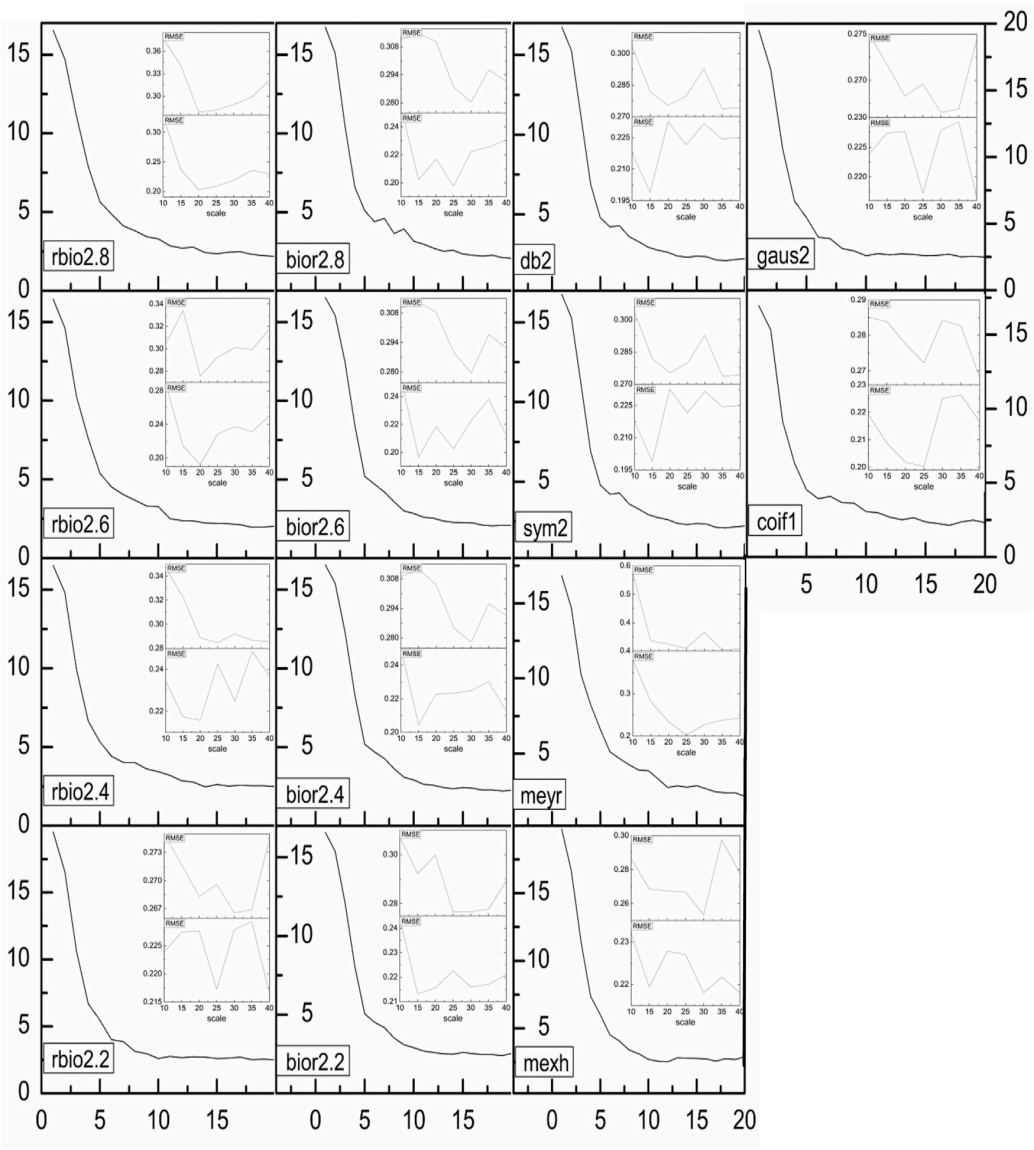
The relationship between Factors and PRESS values for the regression analysis of the signals reconstructed by different WT. The upper is the RMSE for prediction, and the lower is the RMSE for calibration.

By using different wavelets, the above method is utilized to resolve the protein signal in the corn dataset (http://www.eigenvector.com/data/Corn/index.html). The results of the regression analysis for this signal are shown in Table 1. Factor for partial least square analysis is selected by the predicted residual error sum of square (PRESS) values. The relationship between Factors and PRESS values for the regression analysis of the signals reconstructed by different WT are shown in Figure 6. With the gradually increasement of the factor value from 1 to 20 by step 1, if the ratio between the present PRESS value and the former PRESS value is more than 0.9, the former factor value is used to construct regression model.

**TABLE 1:** The results of the regression analysis for the protein NIR signal.

Methods	Wavelets	Data: Corn/Protein				
The proposed method		Factor	RMSEC(Selected a)	R_c_	RMSEP	R_p_
	Rbio2.2	6	0.2161 (15)	0.8985	0.2249	0.9125
	Rbio2.4	6	0.2185 (20)	0.8959	0.2212	0.9166
	Rbio2.6	7	0.2017 (20)	0.9132	0.1938	0.9349
	Rbio2.8	7	0.2095 (20)	0.9055	0.2153	0.9166
	Bior2.2	6	0.2214 (15)	0.8929	0.2533	0.8840
	Bior2.4	5	0.2562 (15)	0.8536	0.2691	0.8669
	bior2.6	5	0.2574 (15)	0.8521	0.2706	0.8657
	Bior2.8	6	0.2201 (25)	0.8941	0.2345	0.9082
	Mexh	8	0.2038 (30)	0.9121	0.2279	0.8989
	Meyr	6	0.2295(25)	0.8881	0.2099	0.9189
	Sym2	6	0.2139 (15)	0.9002	0.2300	0.9095
	Db2	6	0.2139 (15)	0.9002	0.2300	0.9095
	Coif1	6	0.2153 (25)	0.9004	0.2248	0.9069
	Gaus2	6	0.2260 (40)	0.8882	0.2587	0.8602
pls	0.2458					
UVE-pls	0.2349					

RMSEC is the root mean square error (RMSE) value for calibration. Selected *a* is the selected WT scale for modeling. The scale *a* value that is corresponding to the minimum RMSEC is selected when *a* changes from 10 to 40 by step 5. R_C_ is the corresponding coefficient for calibration, and R_P_ is the corresponding coefficient for prediction. We can also easy to compare the method with PLS from the results in the Table.

**FIGURE 7:**
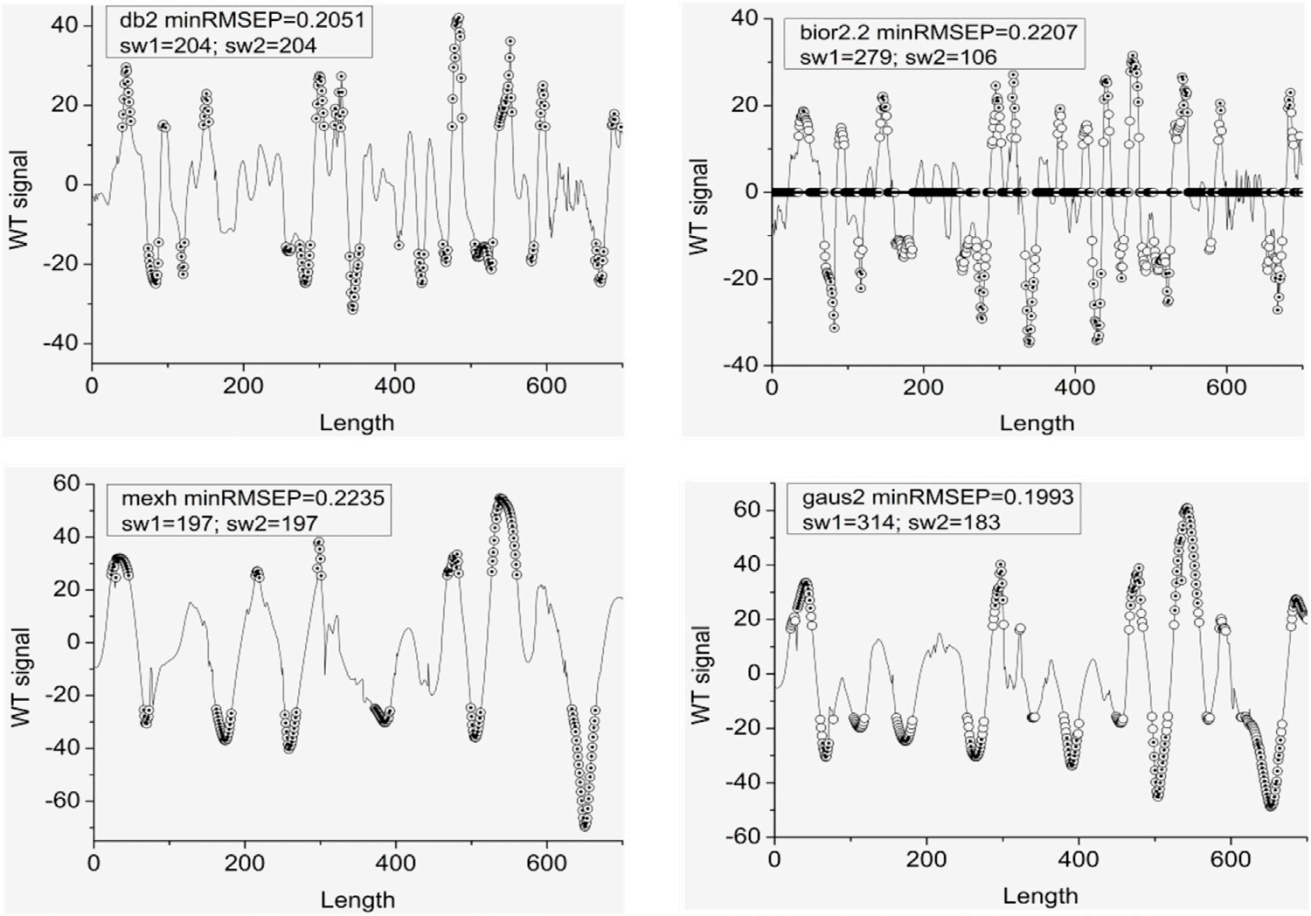
Some select valuable signal points by UVE after WT. minRmsep is the minimum RMSEP value after 100 times UVE. sw is the selected spectrum point number.

UVE is utilized to select valuable signal points after WT, and some selected results are shown in Figure 7 (others in supplementary figures). The curves in Figure 7 are the WT signals, circles are the selected signal points which can generate the minimum PRESS value by UVE for 100 times. If a signal point is selected in all UVE repetitions, a dot is set in the circle. From the results, the peaks and troughs in the WT signal are the valuable information. As mentioned above, the selected troughs in the complex NIR signal may contain some important information.

## Conclusion

The proposed valuable information extraction method can effectively extract the maximum valuable information from NIR signal. All the information in the sub signals of the simulated one are successfully resolved by the method. By resolving actual protein dataset, the detail information in it is totally emerged. After further UVE study, obviously comparable results are obtained. The method will be very helpful for the relative researchers.

## Data Availability

Publicly available datasets were analyzed in this study. This data can be found here: http://www.eigenvector.com/data/Corn/index.html.
