# Supplementary material for: How to Resolve the Maximum Valuable Information in Complex NIR Signal: A Practicable Method Based on Wavelet Transform
Source: Front Chem. 2022 Apr 7;10:812567. doi: 10.3389/fchem.2022.812567 (PMC9021636; doi:10.3389/fchem.2022.812567)
Supplement: Supplementary file 1 [file DataSheet1.PDF]

How to resolve the maximum valuable information in complex NIR signal: a practicable method based on Wavelet Transform

Jing Chen, Xiaoquan Lu*

* Corresponding author.
* Corresponding author.

Supplementary Figures. Some select valuable signal spots by UVE after WT.

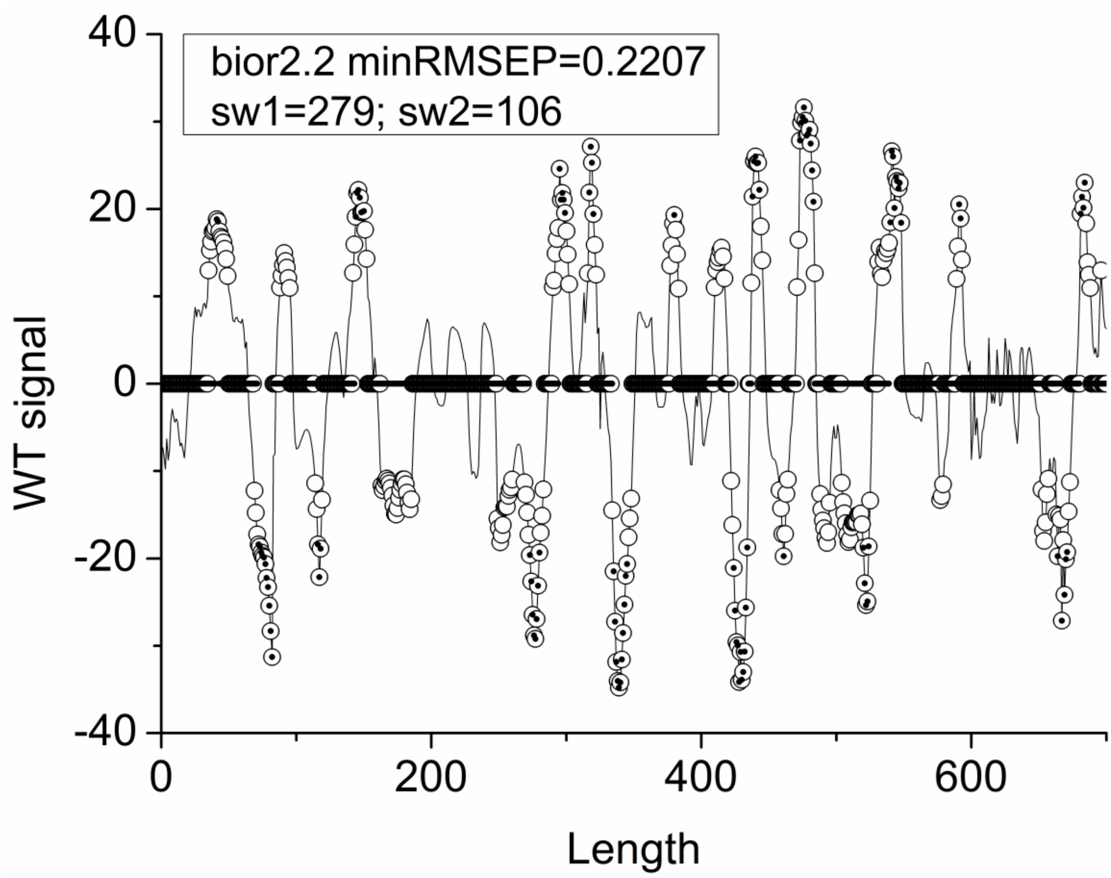

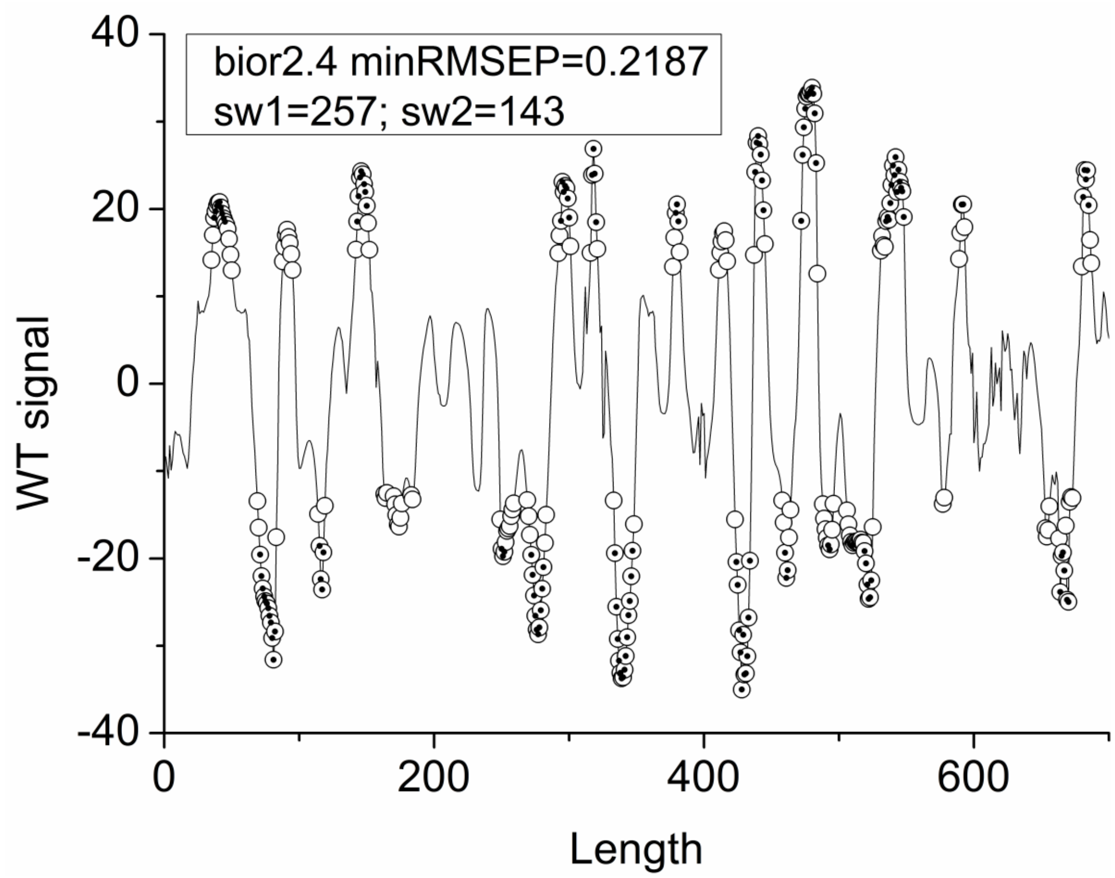
bior2.4 minRMSEP=0.2187
sw1=257; sw2=143
WT signal
Length
40
20
0
-20
-40
0
200
400
600

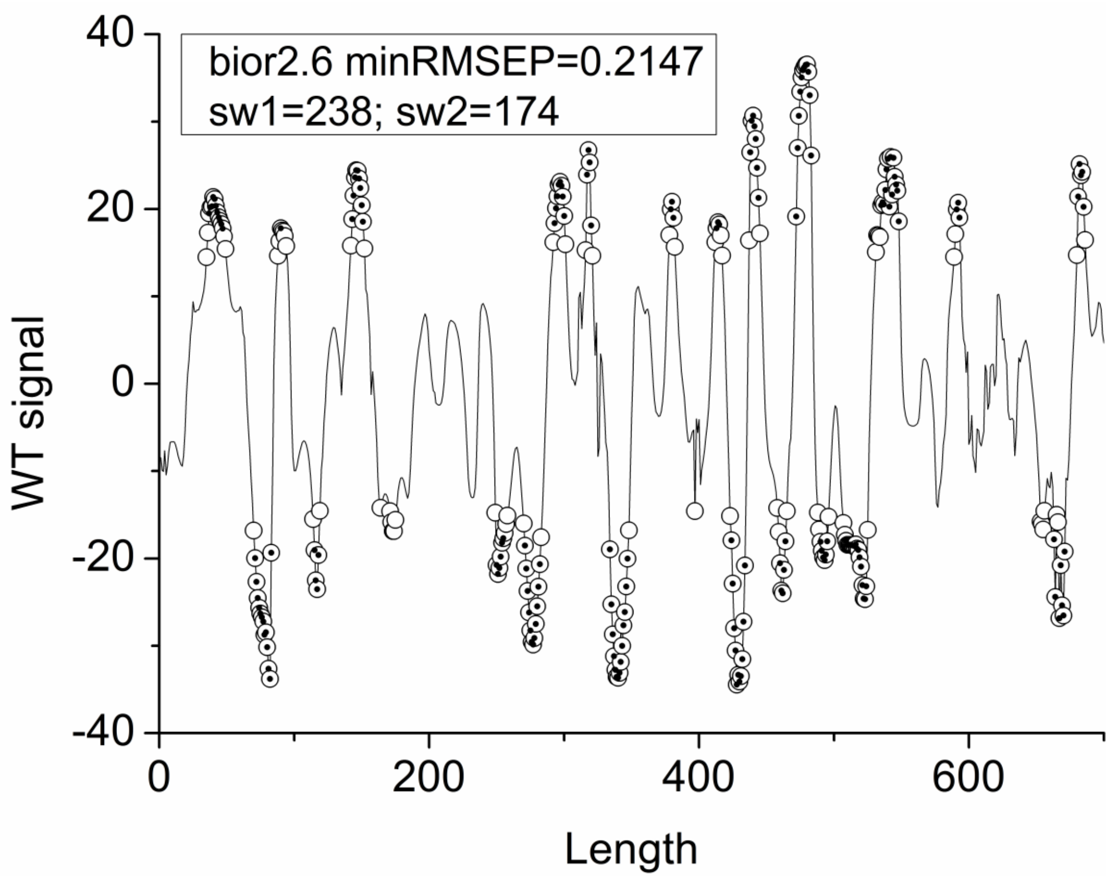
bior2.6 minRMSEP=0.2147
sw1=238; sw2=174
WT signal
Length
40
20
0
-20
-40
0
200
400
600

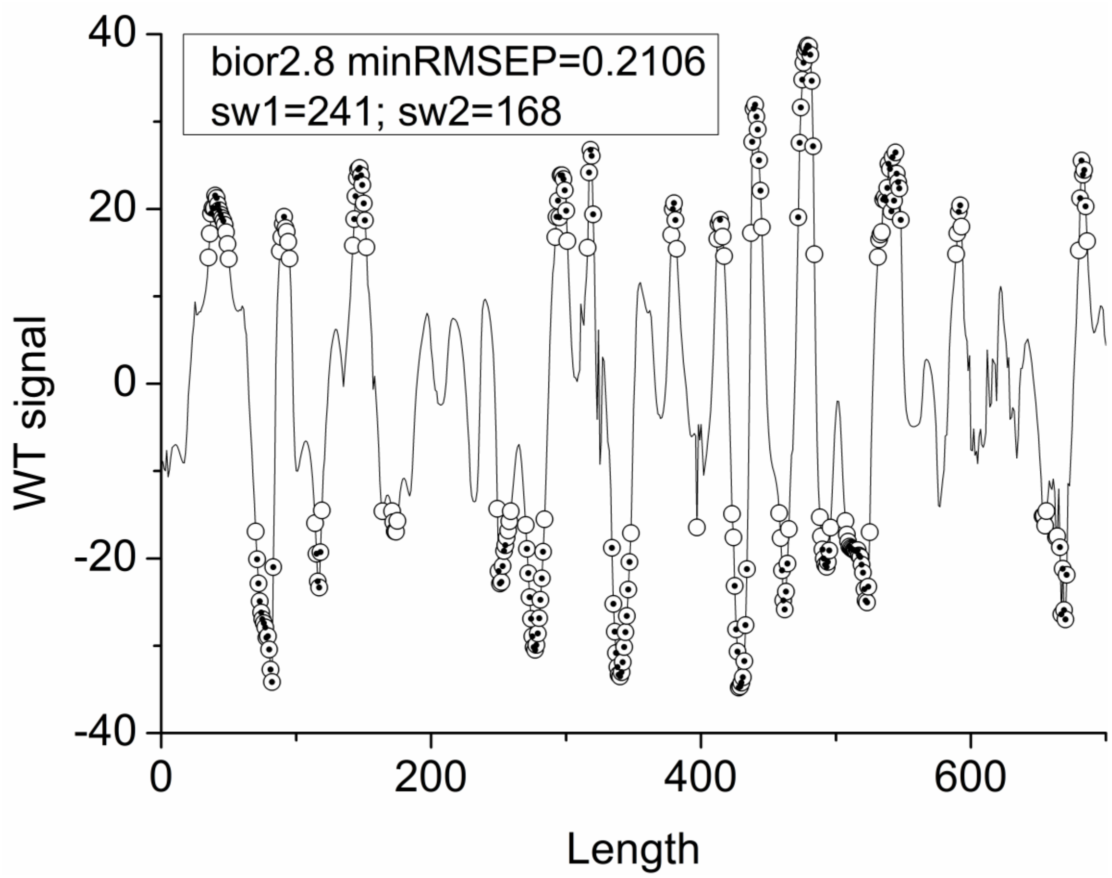
bior2.8 minRMSEP=0.2106
sw1=241; sw2=168
WT signal
Length
40
20
0
-20
-40
0
200
400
600

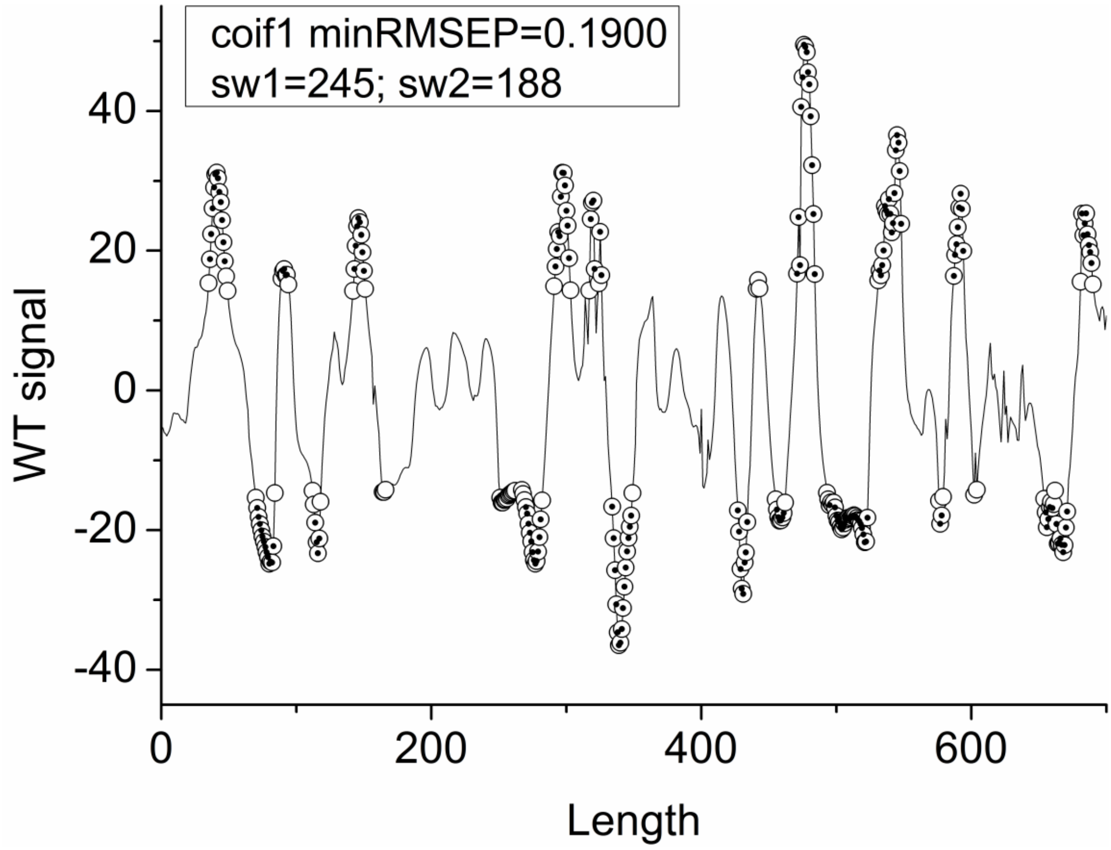

coif1 minRMSEP=0.1900
sw1=245; sw2=188
40
20
0
-20
-40
WT signal
0
200
400
600
Length

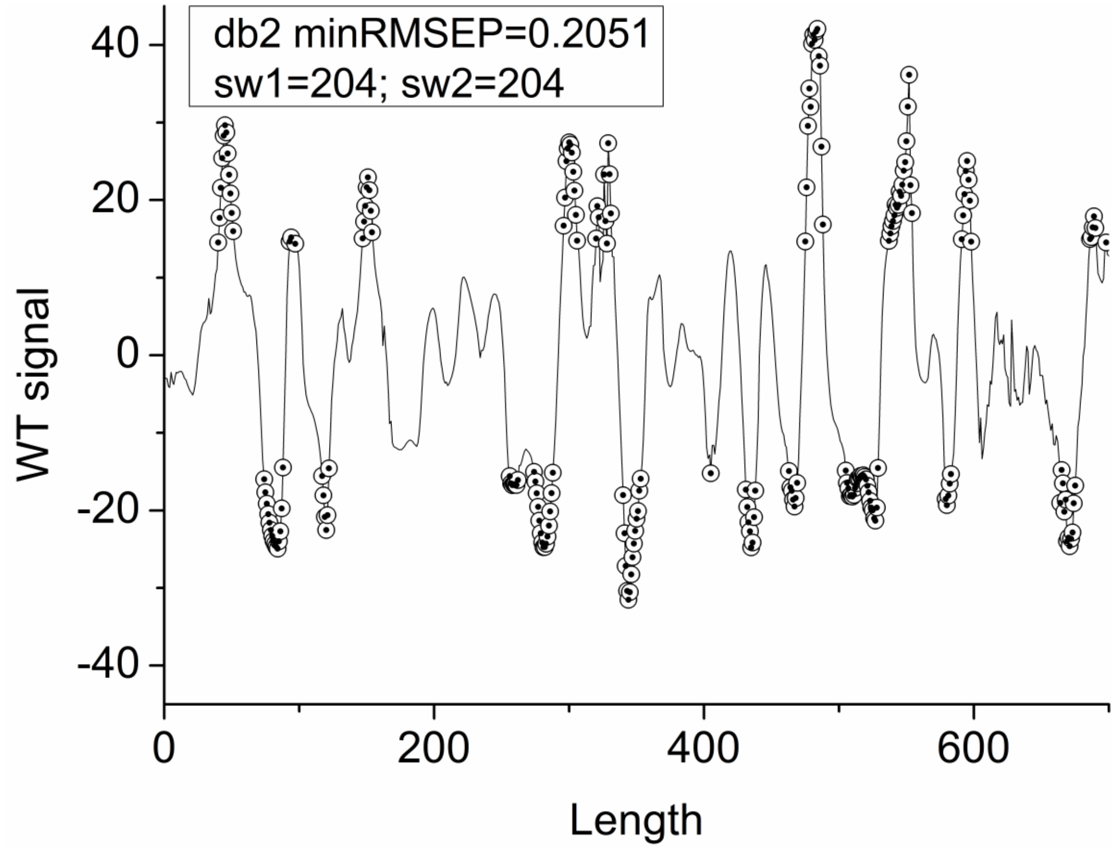
db2 minRMSEP=0.2051
sw1=204; sw2=204
WT signal
Length
40
20
0
-20
-40
0
200
400
600

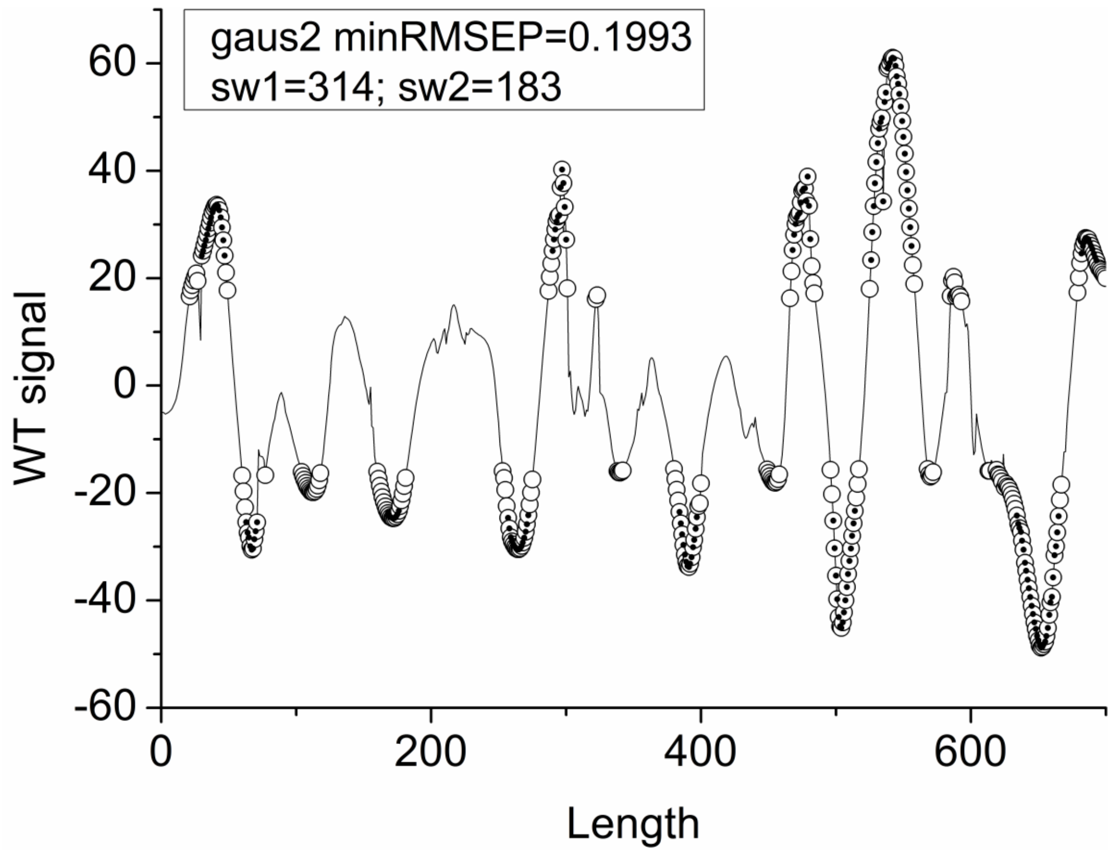

gaus2 minRMSEP=0.1993
sw1=314; sw2=183
WT signal
Length
60
40
20
0
-20
-40
-60
0
200
400
600

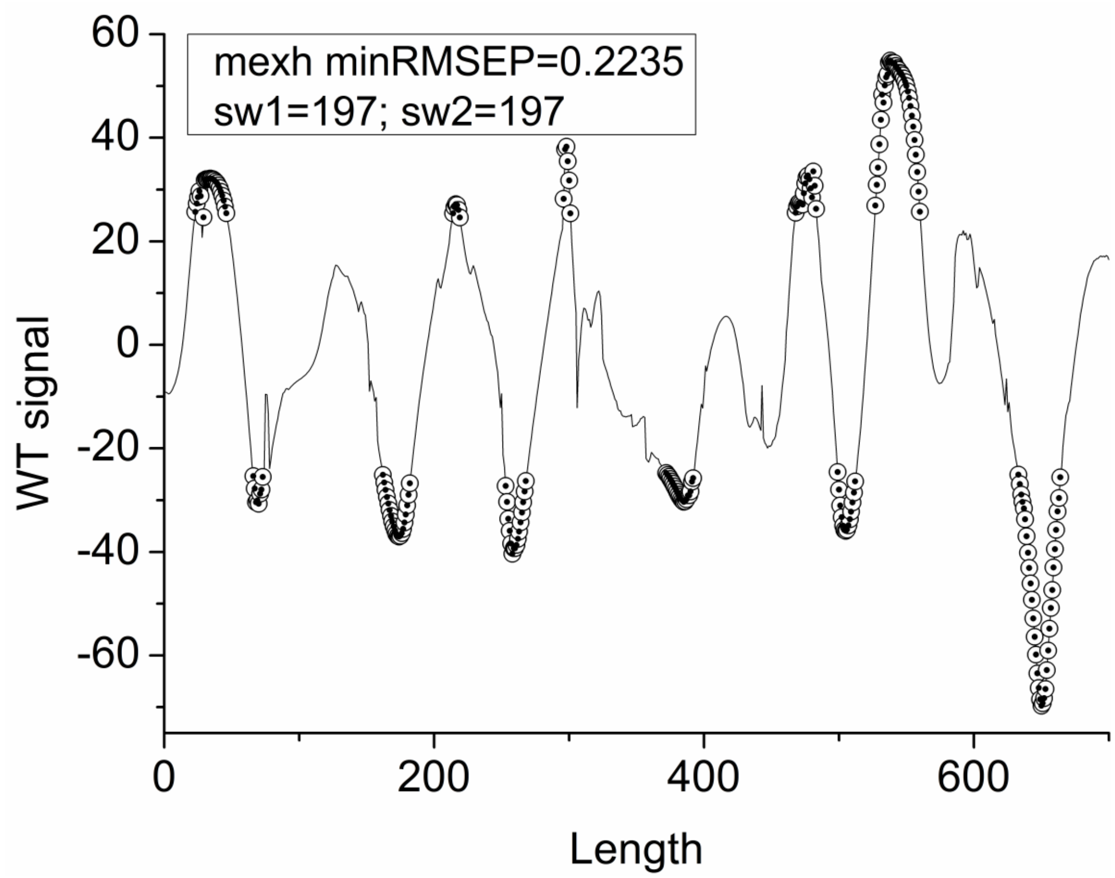

mexh minRMSEP=0.2235
sw1=197; sw2=197
60
40
20
0
-20
-40
-60
WT signal
0
200
400
600
Length

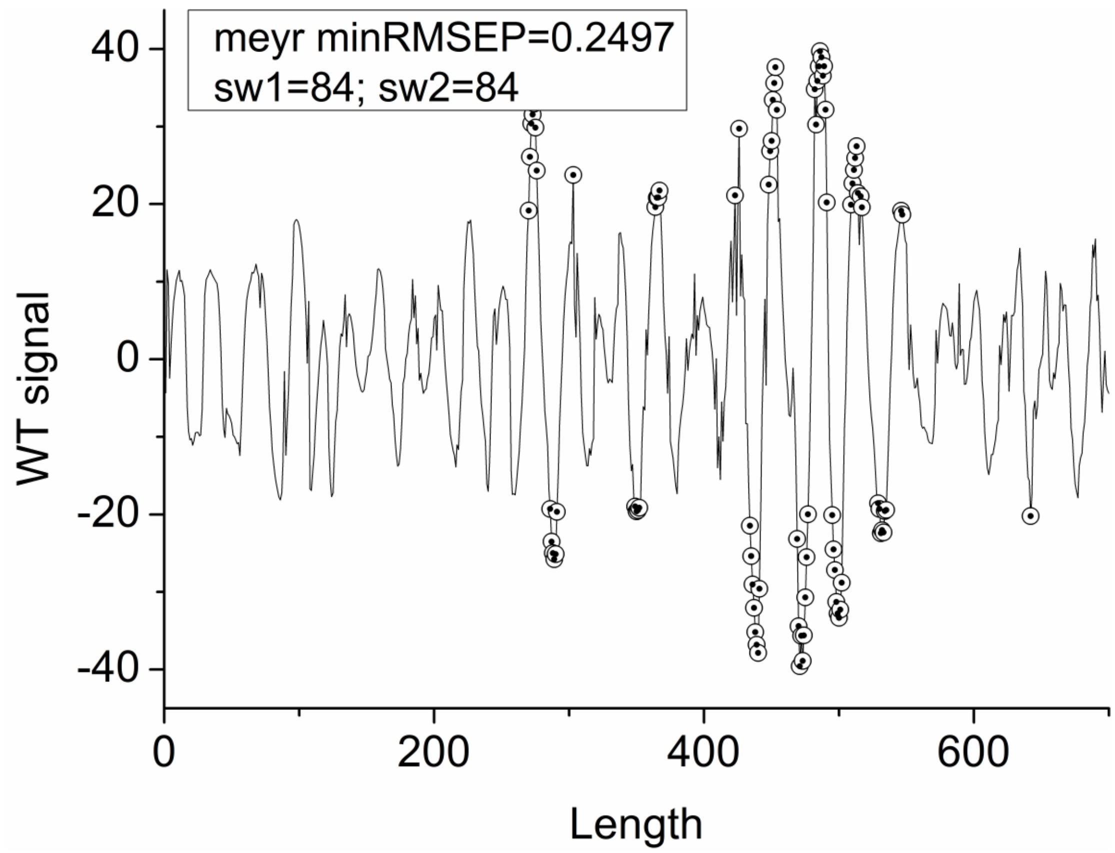

meyr minRMSEP=0.2497
sw1=84; sw2=84
WT signal
Length
40
20
0
-20
-40
0
200
400
600

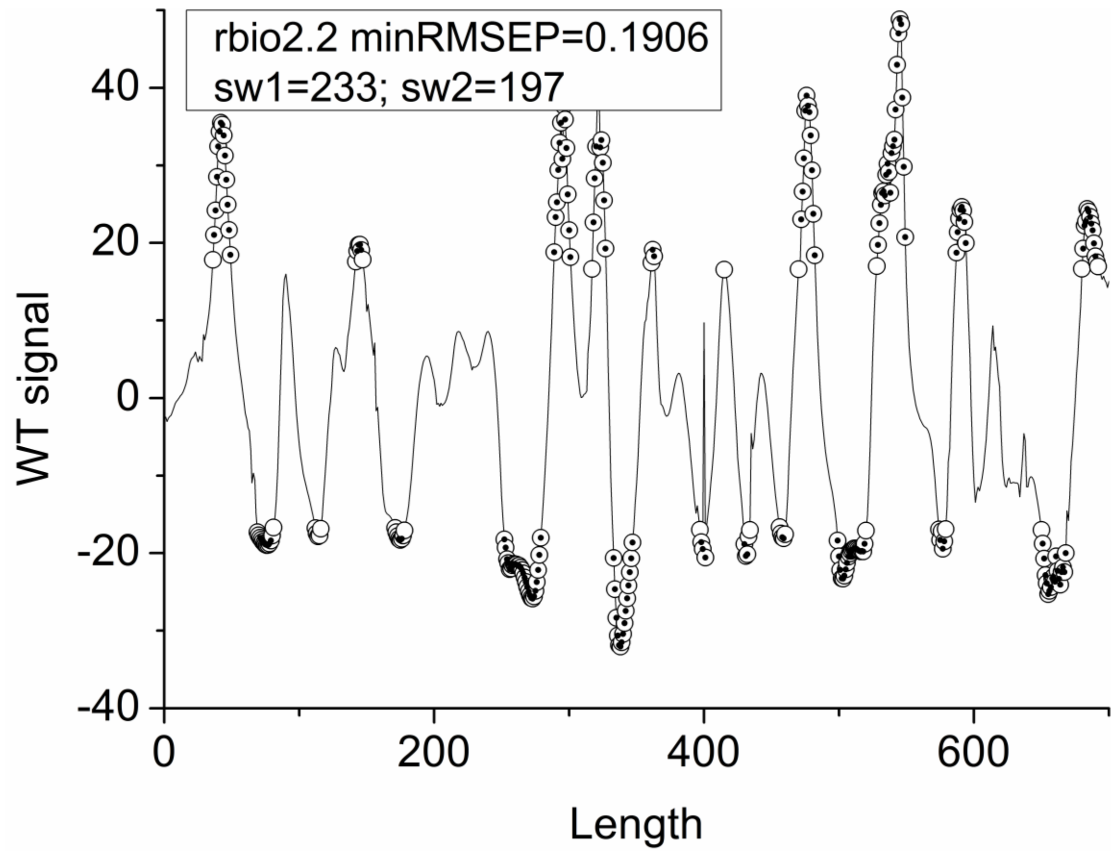
rbio2.2 minRMSEP=0.1906
sw1=233; sw2=197
WT signal
Length
40
20
0
-20
-40
0
200
400
600

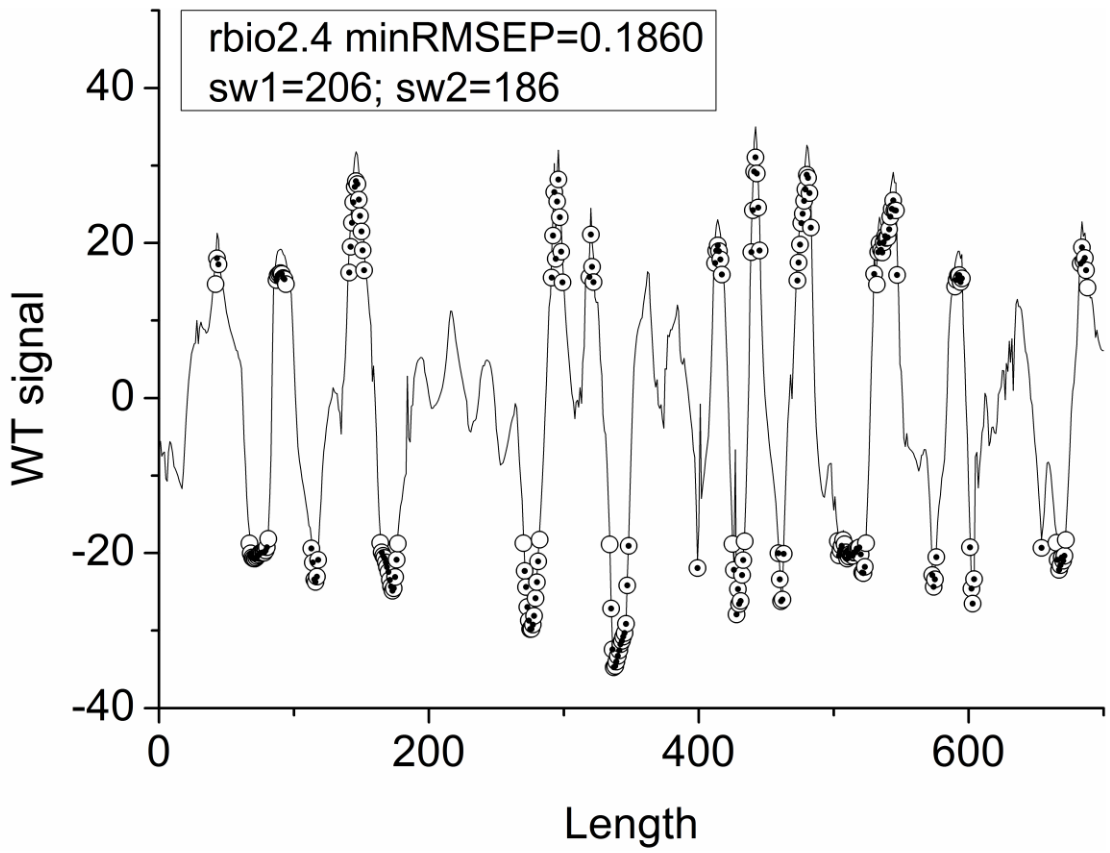
rbio2.4 minRMSEP=0.1860
sw1=206; sw2=186
WT signal
Length
40
20
0
-20
-40
0
200
400
600

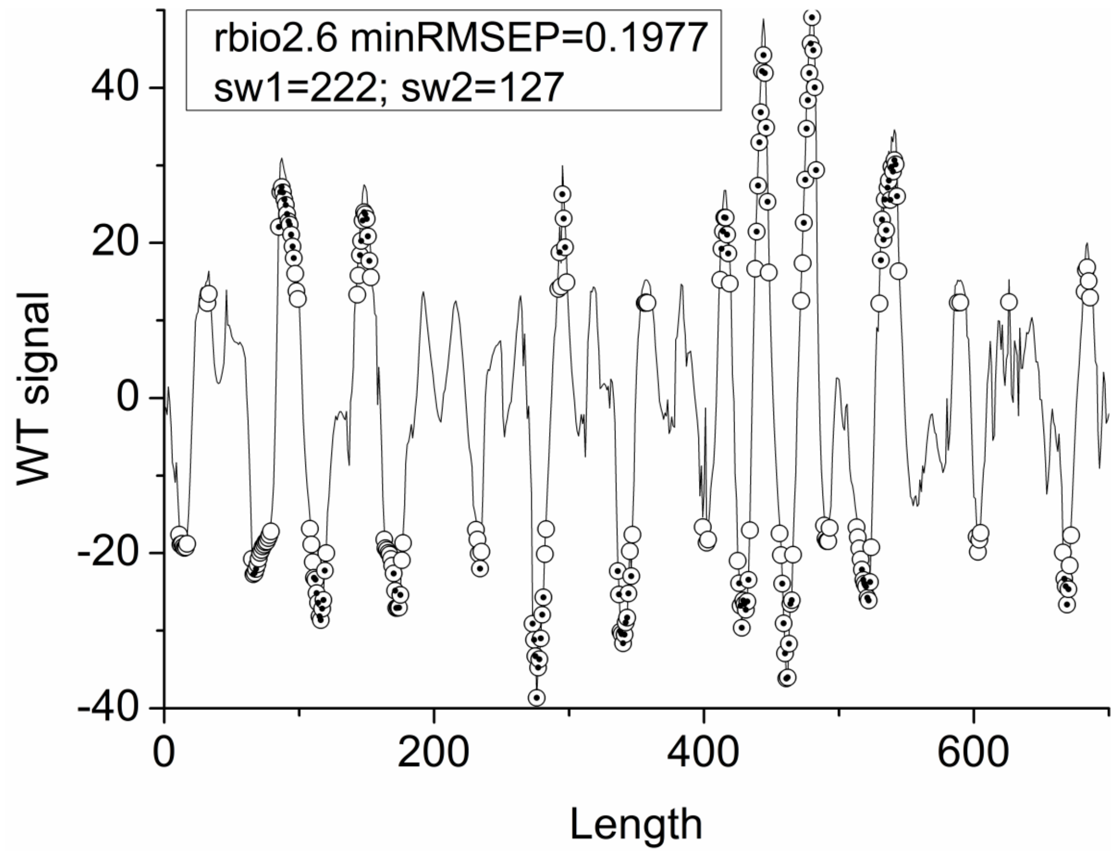
rbio2.6 minRMSEP=0.1977
sw1=222; sw2=127
WT signal
Length
40
20
0
-20
-40
0
200
400
600

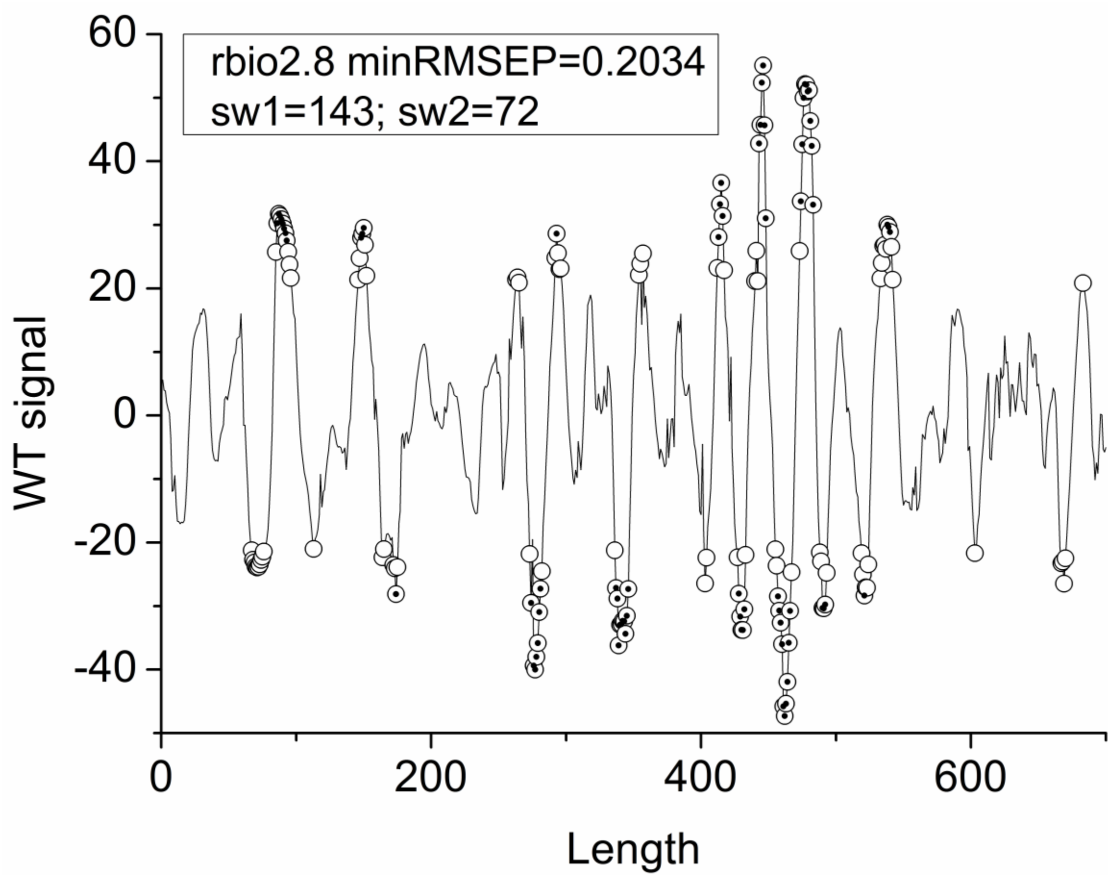

rbio2.8 minRMSEP=0.2034
sw1=143; sw2=72
WT signal
Length
60
40
20
0
-20
-40
0
200
400
600

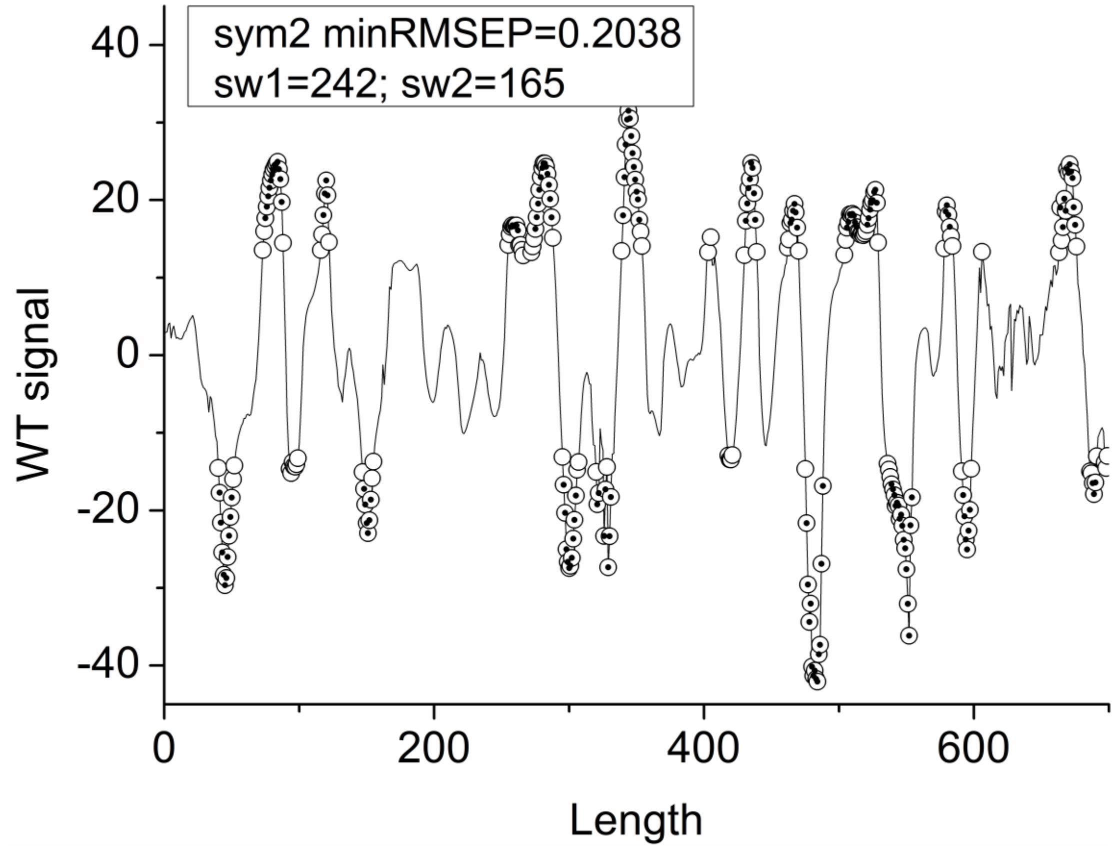
sym2 minRMSEP=0.2038
sw1=242; sw2=165
WT signal
Length
40
20
0
-20
-40
0
200
400
600
